# Low Cost and Compact FMCW 24 GHz Radar Applications for Snowpack and Ice Thickness Measurements

**DOI:** 10.3390/s20143909

**Published:** 2020-07-14

**Authors:** Patrick Pomerleau, Alain Royer, Alexandre Langlois, Patrick Cliche, Bruno Courtemanche, Jean-Benoît Madore, Ghislain Picard, Éric Lefebvre

**Affiliations:** 1 Centre d’Applications et de Recherches en Télédétection (CARTEL), Université de Sherbrooke, Sherbrooke, QC J1K 2R1, Canada; Patrick.Pomerleau@USherbrooke.ca (P.P.); Alexandre.Langlois2@USherbrooke.ca (A.L.); Patrick.Cliche@USherbrooke.ca (P.C.); BCourtem@Ubishops.ca (B.C.); jean-benoit.madore@usherbrooke.ca (J.-B.M.); 2 Centre d’Études Nordiques, Québec, QC G1V 0A6, Canada; 3 Institut des Géosciences de l’Environnement (IGE), CNRS Université Grenoble Alpes, 38 058 Grenoble, France; ghislain.picard@univ-grenoble-alpes.fr (G.P.); eric.lefebvre@cnrs.fr (É.L.)

**Keywords:** Frequency-Modulated Continuous-Wave (FMCW) radar, lake ice thickness, snow water equivalent, snow density, snow wetness, snow monitoring, snow boreal forest, subarctic snow taiga, Arctic snow tundra, Antarctica

## Abstract

Monitoring the evolution of snow on the ground and lake ice—two of the most important components of the changing northern environment—is essential. In this paper, we describe a lightweight, compact and autonomous 24 GHz frequency-modulated continuous-wave (FMCW) radar system for freshwater ice thickness and snow mass (snow water equivalent, SWE) measurements. Although FMCW radars have a long-established history, the novelty of this research lies in that we take advantage the availability of a new generation of low cost and low power requirement units that facilitates the monitoring of snow and ice at remote locations. Test performance (accuracy and limitations) is presented for five different applications, all using an automatic operating mode with improved signal processing: (1) In situ lake ice thickness measurements giving 2 cm accuracy up to ≈1 m ice thickness and a radar resolution of 4 cm; (2) remotely piloted aircraft-based lake ice thickness from low-altitude flight at 5 m; (3) in situ dry SWE measurements based on known snow depth, giving 13% accuracy (RMSE 20%) over boreal forest, subarctic taiga and Arctic tundra, with a measurement capability of up to 3 m in snowpack thickness; (4) continuous monitoring of surface snow density under particular Antarctic conditions; (5) continuous SWE monitoring through the winter with a synchronized and collocated snow depth sensor (ultrasonic or LiDAR sensor), giving 13.5% bias and 25 mm root mean square difference (RMSD) (10%) for dry snow. The need for detection processing for wet snow, which strongly absorbs radar signals, is discussed. An appendix provides 24 GHz simulated effective refractive index and penetration depth as a function of a wide range of density, temperature and wetness for ice and snow.

## 1. Introduction

In northern regions where communities rely on winter transport for their food and fuel supply, safety on ice roads is a major concern. Improved methods for monitoring ice thickness are important in the context of climate warming impacts on the cryosphere. Between 1991 and 2000, an average of 27 snowmobilers died each year from breaking through the ice of frozen lakes and rivers [1]. With increasingly short winters producing thinner ice [2], this number could grow. Furthermore, ice jams and the associated flooding, as well as upstream breakup on rivers, can cause significant infrastructure damage and increase costs related to civil security [2]. Improving our capabilities to monitor ice cover evolution will allow us to optimize various river operations, reduce uncertainty in the occurrence of ice jams and upstream breakup of ice cover, and thus mitigate potential damage while improving ice transport safety. Accurate ice thickness information is also needed to manage safety in recreational winter sports that take place on ice, such as skating on frozen lakes and natural rinks (canals), ice fishing, snowmobiling and cross-country exploration (see charts of safe loads for clear solid ice: http://www.redcross.ca).

From a snow perspective, variability in snow water equivalent (SWE), i.e., the snow mass on the ground, is a key parameter in hydrology (modeling and flood forecasting, [3,4]), climate change impact monitoring [2,5,6,7,8,9] and northern hydropower and water supply management [10]. However, while snow depth is commonly reported in operational weather station monitoring [5,11], this is not the case for SWE monitoring (see the review paper from [12]). While several SWE networks exist in northern regions, such as the Western United States [13], Canada [14,15,16], Russia [17], Finland [18] and the French Alps [19], the availability of these data remains limited (some are commercial data) and sparse. Across the Arctic, continuous SWE measurements are virtually nonexistent, yet snowmelt can contribute up to 33–50% of river flow of arctic watersheds [20].

Based on different types of waveforms, radar systems can be categorized into impulse radar [21], such as ground-penetrating radar (GPR) [22,23,24,25] and the more commonly used continuous-wave (CW) and frequency-modulated continuous-wave (FMCW) radar (reviewed in [26,27]). One can define five main FMCW radar specifications that are important to monitor snow and ice: The radar central frequency and its bandwidth that is scanned, the polarization, the antenna footprint (beamwidth), and radar size. The frequency (wavelength) used specifies the penetration depth, while the bandwidth specifies the distance resolution (the wider the bandwidth, the lower the resolution). The first and most common FMCW radar was in the X-band (10 GHz), operating over 8–12 GHz [28,29], providing a vertical resolution in the order of 3 cm. L-Band FMCW radar (1.12–1.76 GHz) allows higher penetration but suffers from reduced resolution [30]. An L/C-band (2–8 GHz) was also used to successfully retrieve snow depth [31] and C/Ku (8–18 GHz) large wideband FMCW radars are capable of detecting crusts as thin as 0.2 mm within the snowpack [32]. Koh et al. [33] demonstrated the improvement for studying a wide range of snow pack conditions using three different frequency ranges (C-, X-, and Ka-bands). Recently, under NASA’s initiative, researchers deployed with success a 2–16 GHz ultrawideband airborne FMCW radar for measurements of snow thickness over sea ice in polar regions, capable of achieving a resolution of less than 1 cm [34]. The operation frequencies of commercial radar systems are now available at higher frequencies. For instance, Ka-band (24 GHz), which is used in this study, and W-band (77 GHz) have been adopted in automotive radar systems [27]. Despite issues such as high path loss, higher operation frequency and smaller wavelength, these bands improve the sensitivity and resolution of radar systems [26].

Most of these works are based on basic linearly-polarized (LP) antennas in radar systems. The polarization of an antenna plays an important role in a radar system’s performance in terms of its resolution, accuracy, and sensitivity. Circular polarization (CP) is advantageous because it mitigates the Faraday rotation effect, is independent of orientation between transmitter and receiver antennas and has decreased multi-path interferences [35]. Moreover, polarization measurement can provide additional information on ice and snow microstructure, such as insights into ice and snow crystal type [36].

In terms of the radar footprint, which is specified by the beamwidth at −3 dB, a directive antenna is recommended, since a wider beamwidth potentially degrades system performance by receiving more noise from the environment. One of the advantages of the FMCW radar technique is that reasonable-sized horn antennas provide a higher signal-to-noise ratio (SNR) when compared to an identical system using a wide-beamwidth antenna. Recently, radar systems have been miniaturized and integrated onto a printed circuit board (PCB), thanks to the advance of high frequency integrated circuits (IC) and monolithic microwave integrated circuits (MMIC) [27].

The last main important characteristics of a radar system are its size and weight. A very compact and light system including the hardware device facilitates a variety of operating modes, especially in the field, in mountainous regions or on remotely piloted aircrafts.

The purpose of this paper is to introduce and evaluate the performance of a commercially available low-cost and compact K-band (23–25.5 GHz) frequency-modulated continuous-wave (FMCW) radar system for freshwater ice thickness and snow mass measurements.

High frequency FMCW radar sensors have become increasingly popular because of their functionality, portability, accuracy, penetration capability (depending on frequency) and low cost (under US$1000) [27,29]. This increased popularity and low cost are due in part to the fact that K-band (24 GHz) and W-band (77 GHz) CW/FMCW radars have been used in automotive radar systems for more than ten years [27].

Using FMCW radars for snow and ice studies has been well established since the 1970s (e.g., [28,37,38,39]; reviewed in [29]). More recently, multifrequency systems [40] and imaging FMCW synthetic aperture radar [41,42] have been shown to be very useful. Recent studies have shown improvements in sensors and retrieval of snow depth measurements [30,34,43], avalanche studies [44,45], snow stratigraphy based on FMCW echo analysis [32,46] or FMCW-based tomography [47], and ice thickness monitoring [48,49]. Rodriguez et al. [50] described a light and compact home-made FMCW, but it is not widely available. The sensor we describe here is the IMST sentire™ FMCW K-band 24-GHz radar module commercialized by IMST (Kamp-Lintfort, Germany) [51]. 

The innovative aspect of this paper is to demonstrate the extent to which the IMST FMCW radar we used in this study can be used for snow and ice measurements (it was initially developed for operational automotive applications). This sensor is a low cost miniaturized ultra-light radar unit, integrated within a compact waterproofed housing. The commercial software interface can be modified to optimize settings for snow and ice characterization. The post-processing has also been personalized to improve the retrievals. Section 2 and Section 3 present a review of the fundamentals and a description of the radar, respectively. Section 4 presents samples of the radar performance tests collected in automatic retrieval mode for five different applications (Section 4.1, Section 4.2, Section 4.3, Section 4.4 and Section 4.5). As each application has its own method, for readability we describe the experimental protocols separately for each specific application.

## 2. Fundamentals

The principle of FMCW radar has been well known since the 1970s and was described in detail by [26,29] (and the references included in those papers). We only recall here the main practical principle for its use. This type of sensor emits a wave at variable frequencies centered on a reference frequency. When the radar receives an echo from a target, the frequency difference between the emitted and reflected signals can be measured. Since the frequency change rate is known, it becomes possible to measure the time between the emission and the reception of the echo so that the radar–target distance can be calculated (in a manner similar to impulse radars). 

For a semi-transparent medium such as snow or ice, the radar wave is partly transmitted, attenuated and reflected (backscattered). The received power is related to the dielectric contrast within the medium, as well as the losses due to volume scattering, attenuation, and spreading effects. The total loss, expressed by the extinction coefficient $\kappa_{e}$, corresponds to the sum of the absorption coefficient $\kappa_{a}$ and the scattering coefficient $\kappa_{s}$. In radiative transfer theory, the extinction coefficient is related to the effective medium permittivity $\varepsilon_{eff}$ via Equation (1) [52]:

(1) $$\kappa_{e}=2k_{o}Im\left|\sqrt{\varepsilon_{eff}}\right|$$

where the complex effective permittivity $\varepsilon_{eff}=\varepsilon_{eff}^{\prime }+j\;\varepsilon_{eff}^{\prime \prime }$ includes a real ($\varepsilon_{eff}^{\prime })$ and an imaginary part ($\varepsilon_{eff}^{\prime \prime })$ of the effective medium permittivity, $j=\sqrt{-1}$ and $k_{o}$ represent the free-space propagation constant.$\;\varepsilon_{eff}^{\prime }$ is linked to the wave propagation, while $\varepsilon_{eff}^{\prime \prime }$ is the dielectric loss factor. The transmitted signal in the medium is also partly reflected when large changes in dielectric properties occur. In the simplified case of the boundary between two media of effective permittivity i and i + 1, the magnitude of the reflection at perpendicular incidence is proportional to the elementary reflectivity $r_{i}\;$, given by:

(2) $$r_{i}\sim \frac{\sqrt{\varepsilon_{eff,i}}-\sqrt{\varepsilon_{eff,i+1}}}{\sqrt{\varepsilon_{eff,i}}+\sqrt{\varepsilon_{eff,i+1}}}$$

The radar in FMCW mode measures the transit time of the microwave signal in the medium. This transit time (*t*) is proportional to the thickness (h) of the medium multiplied by the speed (v) of the electromagnetic wave in the medium, such as for a two-time way retrieval, i.e., a propagation time from the transmitter to the receiver:

(3a) $$t=\frac{2h}{v_{x}}=\frac{2hn_{x}^{\prime }}{c}$$

(3b) $$n_{x}^{\prime }{}^{2}=\varepsilon_{x}=\frac{\varepsilon_{x}^{\prime }+\sqrt{\varepsilon_{x}^{\prime }{}^{2}+\varepsilon_{x}^{\prime \prime }{}^{2}}}{2}\;\mathrm{or}\;n_{x}^{\prime }{}^{2}=\varepsilon_{x}^{\prime }\;\mathrm{when}\;\varepsilon_{x}^{\prime \prime }\ll \varepsilon_{x}^{\prime }$$

where $\upsilon_{x}=c/n_{x}^{\prime }$; *c* is the speed of light in air; $n_{x}^{\prime }$ is the effective real refractive index; and $\varepsilon_{x}$ is the effective permittivity of the medium (*x*) for ice *x = i,* water *x = w*, dry snow *x = sd* and wet snow *x = sw*. The real refractive index can be expressed by the real ($\varepsilon_{x}^{\prime })$ and imaginary ($\varepsilon_{x}^{\prime \prime })$ part of the medium, as in Equation (3b). We report all theoretical simulated refractive index values ($n_{x}^{\prime })$ at 24 GHz in Appendix A. 

**Figure 1:**
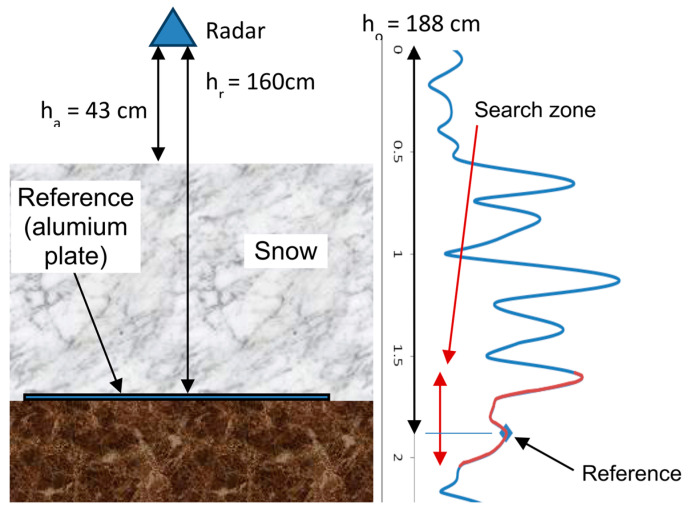
Schematic illustration of the radar retrieval. On the left, the radar is at a height *h_air_* above the snow, that is at a height (*h_r_*) above the ground, given a measured snow depth of *h_s_* = 117 cm. On the right, the measured radar signal expressed in “radar distance” is shown, with a series of peaks linked to different layers within the snowpack. The measured total radar distance is 188 cm between the radar and the last peak (i.e., the bottom). Knowing *h_s_*, one can retrieve the mean snow refractive index or permittivity(Equation (11)).

When pointing the radar vertically downwards at the ice or snow cover (Figure 1), this transit time is converted into the “radar distance” ($h_{radar})$ between the radar and the target through the different media:

(4a) $$h_{radar}=h_{o,air}+h_{o,x}=h_{o,air}+\sum_{l}h_{o,x,l}$$

(4b) $$h_{radar}=h_{air}n_{a}+\underset{l}{\sum }h_{x,l}\sqrt{\varepsilon_{x,l}}=h_{air}+\underset{l}{\sum }h_{x,l}\sqrt{\varepsilon_{x,l}}$$

(4c) $$\sum_{l}h_{s,l}=h_{s}\;\mathrm{or}\;\sum_{l}h_{i,l}=h_{i}$$

where $h_{o,air}$ and $h_{o,x}$ are the radar distance in the air and medium, respectively (*x* = *i, sd,* or *sw*) (Figure 1). If the medium is not homogeneous, it can be considered as consisting of *l* layers Equation (2a). All radar distances can be translated to real distances by considering the refractive index ($n^{\prime }$) or the permittivity ($\varepsilon_{l})$ Equation (4b). As the permittivity of air is $\sqrt{\varepsilon_{a}^{\prime }}=1,\;h_{o,air}=h_{air}$. The radar distance in the medium $\left(h_{o,\;x}\right)$ can thus be expressed as a function of the sum of each snow or ice layer depth $(h_{x,l})\;\mathrm{weighted}\;\mathrm{by}\;\mathrm{the}$ square of the effective permittivity ($\varepsilon_{x,l})$ of the layer (*l*) Equation (4b). The summation over all of the (*l*) layers of the medium corresponds to the total depth of the medium, i.e., the “snow depth” ($h_{s}$) or “ice thickness” ($h_{ice})\;\mathrm{Equation}\;\left(4c\right)$. 

Assuming a homogeneous medium, Equation (4b) for snow becomes:

(5) $$h_{radar,\;snow}\approx \;h_{air}+h_{s}\sqrt{\varepsilon_{s}}$$

where $\varepsilon_{s}$ is the effective permittivity of the whole snowpack and $h_{s}$ is the total snow depth. 

For ice detection, the radar distance to be considered ($h_{radar,ice})$ is the distance between the radar and the bottom of the ice, i.e., at the water interface. Equation (4b) for ice then becomes:

(6) $$h_{radar,ice}\approx \;h_{air}+h_{i}\sqrt{\varepsilon_{i}}$$

where $\varepsilon_{i}$ is the ice effective permittivity and $h_{i}$ is the total ice thickness. 

The system of Equations (4)–(6) includes undetermined problems (more unknowns than equations). It is possible to measure $\varepsilon_{x}$ using an FMCW radar if an independent measurement of snow depth ($h_{s}$) or ice thickness (*h_i_*) is available, or inversely, one can measure snow depth or ice thickness if the mean medium permittivity is known. For wet snow, there is a third variable to be considered, that is, its liquid water content (see Discussion).

### 2.1. Ice Thickness Derivation

For the freshwater ice thickness measurement, the problem (Equation (4)) is solvable, assuming homogeneous solid pure ice at a given temperature (T), which is known. The commonly accepted value of ice refractive index at the frequency used (24 GHz) is $n_{i}^{\prime }=1.78\pm 0.0035$ for 0 ≤ T ≤ –40 °C, and $n_{i}^{\prime }$ $=\sqrt{\varepsilon_{i}}=\sqrt{3.17}$ [53] (see Appendix A). Due to the significant dielectric contrast between ice and water, typically with a refractive index of $n_{i}^{\prime }=1.78\;\mathrm{and}\;n_{w}^{\prime }=4.83$ at 24 GHz and 0 °C, giving a 46% reflection (Equation (2) from ice to water (see Appendix A), the ice/water interface is generally strongly marked with the FMCW radar used. This leads to the relationship between ice thickness ($h_{i})$ and the measured radar distance between the surface of ice and the bottom ice/water interface, from Equation (6):

(7) $$h_{i}\approx \;\frac{\left(h_{radar,ice}-h_{air}\right)}{1.78}$$

However, lake ice is not always homogeneous [48]. Increased air bubble content in ice decreases ice density and thus relative permittivity of bubbled ice down to 3.0 (–6% in density), depending on the volumetric air bubble content, i.e., the density [54]. Furthermore, snow cover is often present on the ice surface and given the dielectric contrast between snow and ice, typically $n_{s}^{\prime }\approx 1.214\;\mathrm{and}\;n_{i}^{\prime }=1.78\;$(19% reflection), the snow/ice interface is generally detectable with the FMCW radar. 

### 2.2. Snow Water Equivalent (SWE) Derivation

It is possible to measure the snow refractive index ($n_{s}^{\prime }$) using an FMCW radar if an independent measurement of snow depth ($h_{s}$) is available. An SWE estimate can thus be derived: $SWE=h_{s}\times \rho_{s}$, where $\rho_{s}$ is the mean snow density linked to the permittivity for dry snow. We focus here on the SWE (permittivity) derivation because snow depth can easily be measured with another sensor, such as LiDAR or an acoustic sensor, or simply with an avalanche probe. The derivation of snowpack stratification is a more complex problem that is discussed in the Discussion. 

Let us consider a dry snowpack with a total snow depth (*h_s_*) characterized by a mean effective permittivity ($\varepsilon_{sd})$. From Equation (5), we can derive:

(8) $$\varepsilon_{sd}={\left[\frac{\left(h_{radar,snow}-h_{air}\right)}{h_{s}}\right]}^{2}$$

For a given range of frequency, the effective permittivity of dry snow was found to depend almost solely on density, i.e., it does not depend on snow type, grain size or temperature [53,54,55,56,57]. The mean relative snow density ($\rho_{s}=\frac{\rho_{snow}}{\rho_{w}},$ where $\rho_{w}$ is the water density) can be retrieved from the estimated permittivity Equation (8) by solving the relationship defined by [57] for dry snow at frequencies between 0.85 to 12.6 GHz:

(9) $$\varepsilon_{sd}=1+1.7\rho_{s}+0.7\rho_{s}^{2}$$

Sadiku [58] showed the extreme stability of dry snow permittivity between 0.6 to 300 GHz, so it is assumed that the Tiuri et al. [57] relationship Equation (9) is still valid at 24 GHz. Several authors proposed slightly different relationships, such as $\varepsilon_{sd}=1+1.83\rho_{s}$ [59] or Equation (10) [53], the latter giving values on average 3% higher:

(10) $$\varepsilon_{sd}=1+1.5995\rho_{s}+1.861\rho_{s}^{3}\;\mathrm{for}\;0\leq \;\rho_{s}\leq 400{\;\mathrm{kg}\;m}^{-3}$$

$$\varepsilon_{sd}={\left(\left(1-\frac{\rho_{s}}{917}\right)+1.4759\frac{\rho_{s}}{917}\right)}^{3}\;\mathrm{for}\;\rho_{s}>400{\;\mathrm{kg}\;m}^{-3}$$

Here, we used the relationship given in Equation (9) for dry snow permittivity.

The final relationship for estimating the dry snow water equivalent (SWE) from FMCW radar measurements, considering the known snow depth (*h_s_*), is solving Equation (9):

(11) $$SWE=h_{s}\times \rho_{s}=h_{s}\times \frac{-1.7+\sqrt{2.89+2.8\left(\;\varepsilon_{sd}-1\right)}}{1.4}$$

where $\varepsilon_{sd}$ is derived from Equation (8), and $h_{radar,snow}$ is the radar distance between the top and the bottom of the snowpack (Figure 1). Note that Equation (11) is derived from Equation (9) and is only valid for dry snow; Equation (11) is thus not valid for wet snow analysis (see Discussion).

## 3. Sensor Description and Operating Modes

### 3.1. Sensor Description

**Figure 2:**
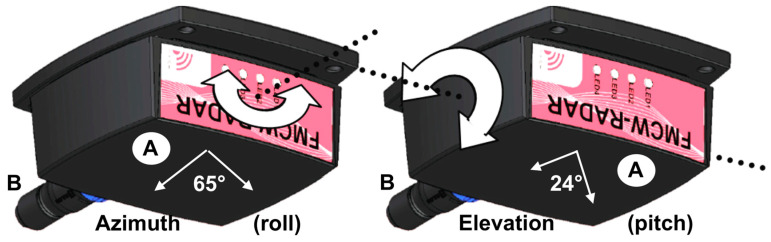
IMST sentireTM Radar Module. A: Radome and antenna position, B: Interface and power supply cable. At one meter above the surface, the radar footprint is about 40 cm.

The low cost compact commercial radar module tested in this study is the K-band 24 GHz IMST sentire™ sR-1200 Series FMCW manufactured by the IMST company, (Kamp-Lintfort, Germany) [51]. The main specifications of this radar are summarized in Table 1 and Table 2 [51] and the hardware is shown in Figure 2. We only used its FMCW mode for our snow and ice characterization application. A simplified link budget is provided in Appendix B.

The radar technology is based on a monostatic FMCW radar system with transmitter and receiver channels and I/Q demodulator [26]. The IMST radar module contains two receiving antennas closed together in one module and fed by one source. The output signal thus consists of four values corresponding to the real and imaginary parts of amplitude, the I and Q channels, respectively, for both antennas. We consider the mean as:

(12a) $$A_{i}=\sqrt{I_{i}^{2}+Q_{i}^{2}}\;\mathrm{and}\;\bar{A}=\frac{A_{1}+A_{2}}{2}$$

In the following, we work on $\bar{A}$ values as the “relative radar signal amplitude”. Note that we could also consider the power signal as:

(12b) $$\begin{array}{ll} P_{I,i}=10\;log{\left(\frac{I_{i}}{I_{o}}\right)}^{2} & P_{Q,i}=10\;log{\left(\frac{Q_{i}}{Q_{o}}\right)}^{2} \end{array}$$

where $I_{o\;}=Q_{o\;}=2^{21}$ is a reference value. We did not use the magnitude of the returned amplitude (power) from reflectors (i.e., ice or snow) because it is much more difficult to interpret than the position of the detected peak.

The maximum possible update rate of measurements depends on many parameters including ramp duration, signal processing time and the amount of transmitted data samples, i.e., the selected maximum detection distance. For our applications, the algorithm we set for measurements optimizes the radar’s parameters, giving a maximum acquisition speed of approximately 15 readings per second. We selected a sampling rate optimization by stretching the signal up to the maximum of 513 values (1/2 of 1024 data samples +1, see Table 1) over a distance of 4 m.

### 3.2. Distance Resolution of the Radar System and Offset 

The theoretical distinction capability between two reflective surfaces, i.e., the distance resolution of an FMCW radar (δh), is only a function of the wave speed (v) and the radar band width (B) [51]:

(13) $$\delta h=\frac{v}{2B}=\frac{c}{2n_{x}^{\prime }B}$$

where v = *c/n*, *c* is the speed of light in air, and $n_{x}^{\prime }$ is the refractive index of the medium. In the air, δh = 6 cm with B = 2.5 GHz (Table 1). In ice, δh is 3.4 cm ($n_{i}^{\prime }$ = 1.78), while in dry snow the radar resolution is in the order of 5 cm at a mean density of 275 kg m^−3^ (down to 4 cm at 500 kg m^−3^).

**Figure 3:**
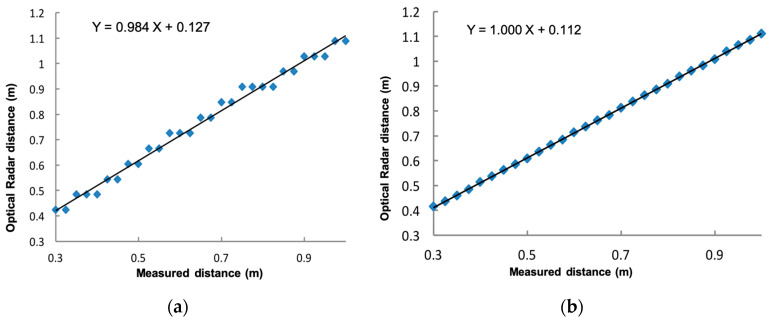
Radar offset determination. (**a**) Results with Tr = 4 ms and padding = 1, showing an apparent stepped sampling effect; (**b**) Results with optimized processing, improving linearity and giving an offset of 11.2 cm.

However, the amplitude and shape of the measured echo after reflection off a target at a relatively short distance (<4 m) depends on the frequency ramp duration (Tr, Table 1) and the sampling mode (padding). A high frequency ramp duration decreases the amplitude of the reflected echo. Setting the padding too low (e.g., to 1) produces a flatter peak than when padding is set to 8, leading to a poorer detection of the true peak in the reflected radar echo. It is thus possible to improve the signal definition (“apparent resolution”) by optimizing these settings (see Figure 2). We also found that using interpolation techniques in order to find the true maximum between measured neighboring points increases the measurement accuracy. The true maximum near the measured maximum must be found in order to estimate the true distance between the radar and the target. Our best results were achieved by setting Tr = 1 ms and padding to 8. The method that improves the search for the true maximum is to perform a weighted average of distances between measured points, taking the maximum amplitude and its two immediate neighbors to obtain the intermediate position corresponding to this true maximum (Figure 3).

The last aspect to consider is the offset in radar echo, which is hardware specific. We had to calibrate each model by measuring the echo for variable distance between the radar and a fixed target. Figure 3 illustrates such a calibration process, showing that, in this case, the systematic bias in the measured signal is 11.2 cm with a precision (standard deviation) of ±0.2 cm.

**Figure 4:**
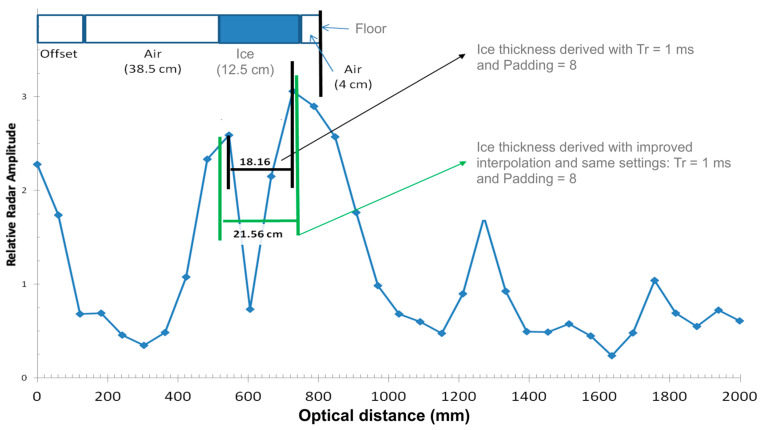
Laboratory test performance on improved processing of FMCW radar signal to estimate ice thickness with a 12.5 cm block of ice measured from a distance of 38.5 cm (see text).

In practice, we found that an accuracy of about 2 cm can be achieved for ice thickness estimates over the radar resolution. An example of the improvement is illustrated in Figure 4, where the radar signal is shown in “optical distance” (i.e., distance relative to air). The mean derived ice thickness by setting the radar at Tr = 1 ms and padding = 8 was 18.2 ± 3.4 cm, which corresponds to using Equation (5) to reach 18.2/1.78 = 10.2 cm—lower than the true measured value. By applying the proposed interpolation method between measured maximums, the derived ice thickness is found to be 21.6 ± 0.5 cm, or using Equation (7): 21.6/1.78 = 12.1 cm, which is closer to the true measured value of 12.5 cm (Figure 4).

### 3.3. Penetration Depth

The theoretical penetration depth (δP) of microwave radiation in an absorbing medium is linked to the frequency (ν) (wavelength, λ=c/ν) and its complex permittivity, which is temperature-sensitive. This is defined by [60]:

(14) $$\delta P=\frac{c}{4\pi \nu }\frac{1}{\sqrt{\frac{\varepsilon^{\prime }}{2}}\left({\left(1+{\left(\frac{\varepsilon^{\prime \prime }}{\varepsilon^{\prime }}\right)}^{2}\right)}^{1/2}-1\right)}\approx \frac{\lambda \sqrt{\varepsilon^{\prime }}}{2\pi \varepsilon^{\prime \prime }}$$

The term on the right is an approximation often used when $\varepsilon^{\prime \prime }\ll \;\varepsilon^{\prime }$. Note that the penetration depth corresponds to the distance at which microwave power is reduced to 1/e (e = 2.718) from the ice or snow surface. This is not the maximum detection depth of the sensor.

For a 24 GHz radar through pure ice, if we consider the two-way radar signal time $\delta P_{r}=\delta P/2$, Equation (11) yields $\delta P_{r}\;$= −1.9298E-02 T + 6.0610, where T is the ice temperature (in Kelvin, see Appendix A). Going from 0 to –30 °C, $\delta P_{r}$ increases from 0.80 m to 1.57 m.

The radar penetration depth ($\delta P_{r}$) of dry snow significantly decreases with density, following a power law, which varies with temperature. At T = 0 °C, $\delta P_{r}$ decreases from 6.78 to 4.81, 3.26 and 2.05 m for densities of 150, 200, 275 and 400 kg m^−3^, respectively. For a medium density of 275 kg m^−3^, $\delta P_{r}$ increases from 3.26 to 4.78 and 6.38 cm at T = 0 °C, −20 °C and −40 °C, respectively (see Appendix A). The case of wet snow, which drastically reduces δP , is discussed in Section 5.

### 3.4. Operating Modes

**Figure 5:**
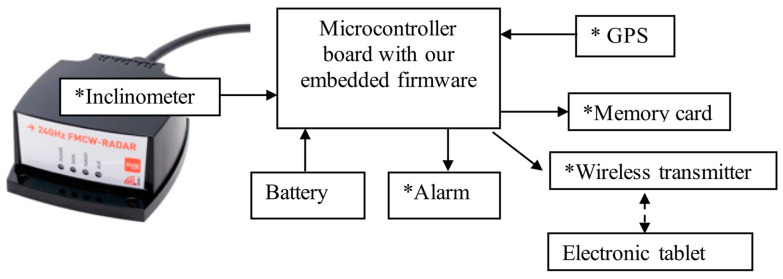
Assembly block diagram for controlling and operating the radar. * Optional, depends on the final application.

The radar is generally controlled and operated with a field tough book or laptop loaded with the software provided by IMST. However, for several applications involving snow or ice monitoring over remote regions, we developed a light compact system with our own controller. The different components of the system, which allow on board signal processing, are described in Figure 5.

The radar is connected to a home-made printed circuit board (PCB) using Arduino^©^ or to a Raspberry Pi^©^ microcontroller and data logger. It is loaded with processing software allowing the automatic calculation of snow and ice thickness. Data are recorded on the memory board and/or sent wirelessly (via Bluetooth) to a smartphone or a tablet for real-time visualization of radar profiles and the measured snow water equivalent (SWE) or ice thickness. The system is powered by a small lithium-ion battery and can be switched to a deep sleep state, consuming less than 5 µW between two series of measurements. An audible or visual alarm can be included to warn if the system detects an ice thickness thinner than a critical value. A GPS receiver can be added to include geospatial information in the saved data but is not necessary for calculating SWE or ice thickness. An inclinometer added to the radar unit ensures that it is parallel to the surface target before beginning a series of measurements, and it filters valid data when the radar is moving.

The low cost of the IMST radar (€800 in 2018) and the use of an Arduino or Raspberry Pi^©^ controller make this extremely reliable system a very cost-effective instrument. All processing is done on board automatically without human interaction. Two operating modes can be distinguished: The manual in situ mode and the continuous autonomous mode. For ice thickness measurements, the processing is the same for both modes with the following sequence: (1) Automatic detection of ice/water interface (last peak); (2) detection of the first peak encountered above the last peak, which corresponds to snow/ice if there is snow or air/ice if there is no snow; (3) detection of the first peak from the radar, which corresponds to the air/snow interface if there is snow, or to the air/ice interface if there is no snow; (4) comparison between (2) end (3) (whether or not there is snow); (5) distance calculation with peak position refinement (see Section 3.2); and (6) ice thickness retrieval. 

For the SWE measurement, the processing sequence is different for the two operating modes, since the snow depth must be known. In manual in situ mode, the sequence is as follows: (1) Manually provide snow depth to the radar; (2) automatic detection of the air/snow interface (first reflection peak); (3) automatic detection of the snow/ground interface (last peak) (see Section 2.2); (4) distance calculation with peak position refinement (see Section 3.2); (5) retrieval of the average (bulk) refractive index; and (6) SWE calculation. In the continuous and autonomous measurement mode (snow monitoring station), the processing sequence only differs for the first step, where the measurement of snow depth is provided by a separate sensor (LiDAR or sonic).

## 4. Results: Radar Performance Tests

We tested the system performance using five operating modes for different applications, first for ice thickness (Section 4.1 and Section 4.2), and then for snow (Section 4.3, Section 4.4 and Section 4.5):

- In situ ice thickness: Manual in situ lake ice thickness measurements recorded by walking on the lake or from a stationary snowmobile. The system was not been tested on a moving snowmobile, although this is possible in a continuous recording mode.
- Ice thickness measurements from a remotely piloted aircraft (RPA) system: A preliminary test was conducted to evaluate the potential of measuring ice thickness with the radar mounted on a RPA.
- In situ SWE: The manual in situ snow water equivalent (SWE) measurement was based on known snow depth value, measured using an avalanche-type snow depth probe.
- In situ snow density: We tested the system in particular conditions in Antarctica to assess temporal snow surface density fluctuations.
- Monitoring SWE: Continuous automatic measurements of SWE evolution during the winter at a weather station. In this case, the radar measurements were combined with a synchronized and collocated automatic snow depth sensor (ultrasonic or LiDAR sensor).

Below, we present examples of results for these applications, showing the performance and limitations of the system. In each case, we describe the experimental protocol and the processing procedure to automatically determine the desired relevant parameter.

### 4.1. In Situ Ice Thickness

#### 4.1.1. Shallow Ice Experiment from A Bridge

**Figure 6:**
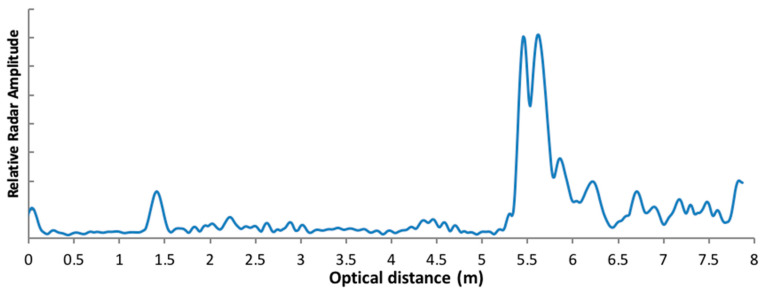
Relative radar signal amplitude (Y axis, starting from distance 0) measured from a bridge over ice at a height of 5.5 m. The width of the two peaks detected around 5.5 m correspond to 9 cm ice thickness.

We first tested the effective resolution of the system for its shallow ice thickness limit. Measurements were performed from a bridge, with the radar pointing downward at approximately 5.5 m above the ice (Magog River bridge, Sherbrooke, QC, Canada, Figure 6). In Figure 6, the first peak at 1.4 m corresponds to an echo of the bridge structure. The distance between the two high peaks around 5.5 m gives an ice thickness estimate of 9 cm. The ice was too thin to be able to safely measure ice thickness exactly within the radar footprint, but ice thickness was confirmed on the riverbank. This experiment remains interesting as it demonstrates the radar’s ability to measure ice thickness near the safe limit of around 10 cm (Canadian Red Cross recommendations, https://www.redcross.ca). 

#### 4.1.2. In Situ Ice Thickness Measurements

A series of radar measurements were acquired along with in situ ice thickness measurements (holes in the ice) for a series of frozen lakes in different cold conditions, with and without snow. The radar was pointed toward the ice at a height of 30 to 50 cm above the surface, with or without snow. Due to permittivity differences between media, the typical profile of relative radar signal amplitudes shows two peaks corresponding to the air/ice and ice/water interfaces without snow cover on ice. In the presence of snow, an additional peak appears before those two peaks, corresponding to the air/snow, snow/ice and ice/water interfaces. An automatic peak detection algorithm was first applied to select all peaks above a threshold to avoid noise. The threshold given by the average of the whole profile gives the best results. We then retrieved the distance between the last peak (usually with the largest amplitude) and the peak before it. From Equation (5), the difference between these peak gives the ice thickness.

**Figure 7:**
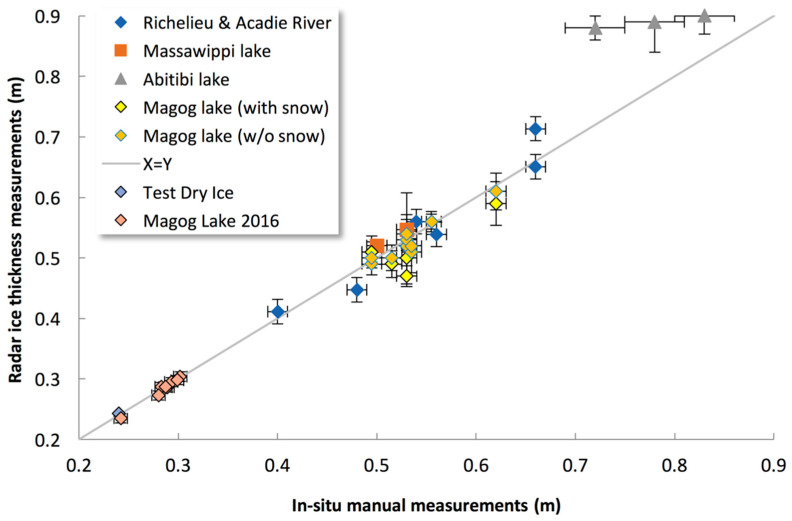
Radar ice thickness measurements compared to in situ manual measurements under frozen conditions. All radar data are from operational automatic retrievals. The horizontal and vertical lines at each point give the in situ and radar precision, respectively.

Figure 7 illustrates the results of fully automatic ice thickness detection. At five different lakes in Québec Province, North-Eastern Canada, we took 35 measurements with collocated holes in the ice, always in cold conditions (dry ice surface). The measurements taken included “test dry ice” experiments (blue diamond symbol in Figure 7) showing deviations from the 1:1 line for some, which may have been caused by several factors such as poor detection of snow depth on the ice, perpendicular misalignment of the radar with the ice surface, or impure ice. Note that one experiment was conducted over ice thickness values of 0.72–0.83 m (Abitibi Lake, Northern Quebec, gray triangle in Figure 7) around (or over, depending on ice temperature) the limit of radar detection. Unfortunately, we observed water lenses in the ice at measured sites that attenuated the signal, i.e., increased the ice refractive index, leading to reduced measured ice thickness (Figure 7). Without these three biased points, the mean accuracy of the radar is confirmed to be 2 cm (root mean square error) with a bias of −0.4 cm (Figure 7).

#### 4.1.3. Limitations of Ice Thickness Detection 

When a thin film of liquid water is on the ice surface, the dielectric contrast between the air/water interface is strong, giving a marked first echo, but with strong absorption underneath as water absorbs the signal (high imaginary part of the permittivity). This leads to a weak, even invisible, echo from the bottom of the ice/water interface, making ice thickness measurements impossible. Another point that must be considered is that we assumed the ice was pure in the operational detection process. That will not be always the case, such as for “white” or “snow ice”, which is a mix of frozen snow and ice caused by the freezing of wet snow on the lake ice’s surface. White ice forms a less dense layer on top of “black” lake ice. As the refractive index of white ice can be significantly lower than pure ice, the apparent thickness measured by the radar is increased.

### 4.2. Ice Thickness Retrieval from Remotely Piloted Aircraft (RPA)

**Figure 8:**
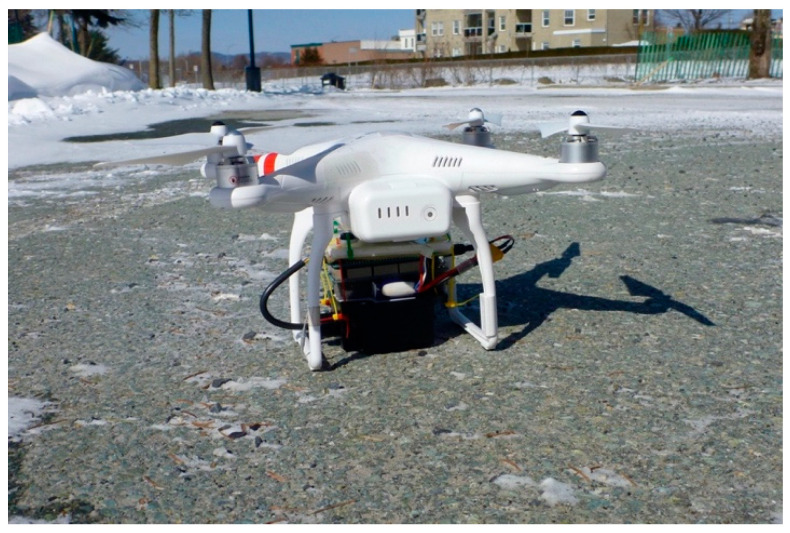
Radar system mounted under a Phantom 2 remotely piloted aircraft (RPA) quadcopter.

A remotely piloted aircraft (RPA) system can be very useful for monitoring thin ice at the beginning of the winter or over a large lake ice area. The compactness and low weight of the radar studied here allows it to be deployed on off-the-shelf commercial RPAs. A preliminary test was conducted in order to evaluate the performance of measuring ice thickness using the radar onboard a Phantom 2 (Figure 8). In this test, the system included a GPS and an inclinometer for recording viewing angle variations (Figure 5). The weight of the radar system, including battery, GPS, inclinometer and the control system (Arduino) was 480 g. The data were recorded on a micro-SD card. 

**Figure 9:**
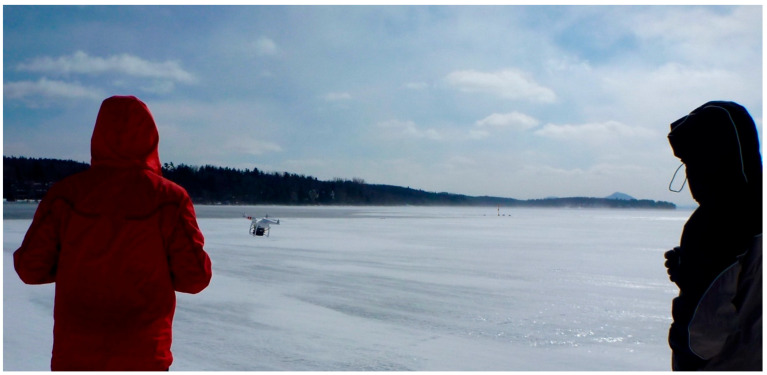
The remotely piloted aircraft in flight. A buoy can be seen in the background where open water starts (ice thickness = 0 cm, stream flow channel in the frozen lake).

Weather conditions during the test flight were challenging, with a mean wind speed of 20 km/h (gusting up to 35 km/h) and air temperatures of −15 °C. Nonetheless, we were able to successfully fly the RPA (Figure 9). A test stationary flight was first carried out at 2.5 m above the ice, giving a radar footprint of 1 m. Four flight lines (L1 to L4) were then followed at a mean altitude of about 5 m (not measured; note that for the IMST Radar Module, the maximum measurement range capability corresponds to $R_{max}=\frac{c\;\left(1024/4\right)}{B}=30.7\;m$). Eight holes were drilled through the ice to measure thickness across the studied area to validate the results. Ice thickness varied from 50 cm to 0 cm near the lake’s edge. 

**Figure 10:**
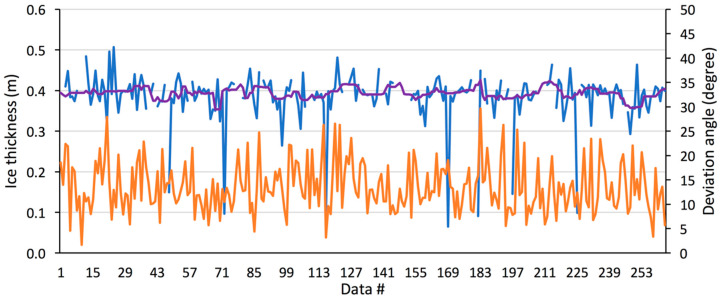
RPA-retrieved radar thickness (blue line, left Y axis), median filtered signal (purple line) and radar viewing angle (orange line, right Y axis) for a stationary flight at 2.5 m above the ice.

An example of data recorded during the stationary flight is illustrated in Figure 8. It shows significant fluctuations in the retrievals (blue line), including strong drop-out values. These values relate to the difficulties in identifying the right echoes of the ice surface, ice bottom or both. Overlaying the synchronous viewing angle record (orange signal in Figure 10) with the measurements revealed the RPA’s relatively important motion variations (pitch, yaw, roll) and the poor correlation between the measured fluctuations in derived ice thickness and the off-nadir viewing angle variation. It was difficult to define an objective data quality threshold. More experiments are needed to better determine the appropriate filters. Here, we applied an arbitrary threshold of 20° to off-nadir viewing angles and smoothed the retrieval with a median mobile filter (purple line in Figure 8).

For the test stationary flight, the mean RPA-based ice thickness was 39.3 cm, compared to the in situ ice thickness of 39 ± 0.5 cm.

**Figure 11:**
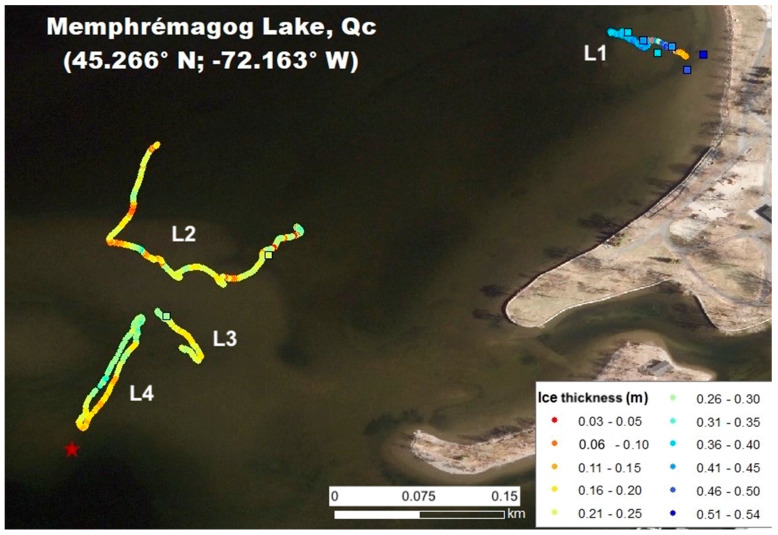
Ice thickness spatial distribution (colors) from in situ measurements (squares) and RPA-based radar data (circles) overlaid on a summer aerial photo. The red star shows the buoy that delimited the open water (see Figure 9). L1 to L4 correspond to the four different RPA flights. The experiment was performed off Pointe-Merry Park, on the right, on Lake Memphremagog, Québec, Canada.

The results for the four flight lines (L1 to L4) are shown in Figure 11. They clearly show that the RPA captured the spatial variation in ice thickness, from 50 cm along L1 on the lake edge (blue points in Figure 11) to about 25 cm along L2 and L3, and to open water (near the buoy, red star in Figure 11). The mean RMSE between the RPA-based and in situ measurements for seven points (no RPA measurements for one validation point) is 12 cm. However, near the lake’s edge at the end of L1, clear disagreements are apparent for two points, where we observed the presence of liquid water trapped in the ice and because the ice extended down to the lake bottom (ice/water interface not detectable). 

Without these two points, the RMSE becomes 1.6 cm with a weak bias of 0.2 cm. This agreement corresponds to the accuracy derived from in situ measurements (previous section). Despite the limited flight time of this test experiment, these results appear very promising. The RPA-based approach allowed ice thickness measurements where it was too thin to support a person’s weight (yellow–red zones in Figure 11).

### 4.3. In Situ Snow Water Equivalent (SWE)

In this section we present a series of tests to evaluate the accuracy of the FMCW system when used in fully automatic mode without any intervention in the retrieval of the measured signal. With the radar pointed toward the snow cover (as in Figure 1), preliminary tests showed that it is sometimes difficult to automatically identify the peak corresponding to the snow/soil interface (P_max_). Knowing the snow depth measured with a snow depth probe, we improved the retrieval by searching for the approximate distance of P’_max_ assuming a mean permittivity value (first guess). We then located the exact position of P_max_ within a search zone around the first guess (red zone in Figure 1) and derived the true mean permittivity and then calculated the SWE value using Equation (8).

**Figure 12:**
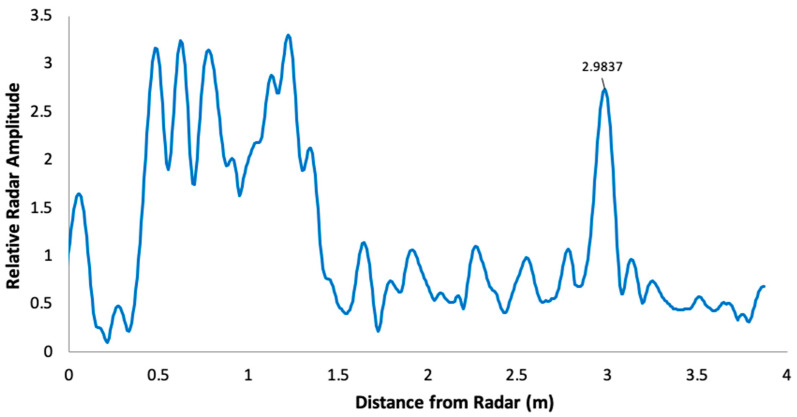
Measured relative radar amplitude signal over dry snowpack (snow depth of 237 cm). The right-most peak, at a distance of 298 cm (snow/plate interface) from the radar, allowed us to estimate the mean snow permittivity when the measured snow depth was known (Equation (8)). Intermediate peaks correspond to layering within the snowpack (see Discussion).

In the following examples, we put a metallic plate on the ground beneath the snowpack to accentuate the snow/soil interface signal, as it increases the snow/plate permittivity contrast. Figure 12 illustrates a measured radar signal over a 237 cm-thick dry snowpack. It is used as an example to describe the automatic retrieval processing. The maximum detection algorithm identified the first peak after 0 as the starting point (position of the radar), the second peak as the air/snow interface and the last peak as the snow/plate interface. The derived radar distance $h_{radar,snow}$ between the top and the bottom of the snowpack allowed the estimation of the mean permittivity when the snow depth was known, and then the snow water equivalent (SWE) was calculated using Equation (11). The radar-based SWE was then compared to the SWE derived from a collocated snowpit with measured density using a 250 cm^3^ (5 cm) density cutter, and with samples weighed using an electronic balance with an accuracy of 0.1 g. The accuracy of the density cutter measurements is about 9% [61] giving an approximate relative SWE accuracy of 11–12%. In Figure 10, from the radar distance estimate (298 cm) and measured snow depth (237 cm), we deduced a 738 mm SWE (Equation (11)), compared to 748 mm from the in situ snowpit measurements (−1.3% difference). 

**Figure 13:**
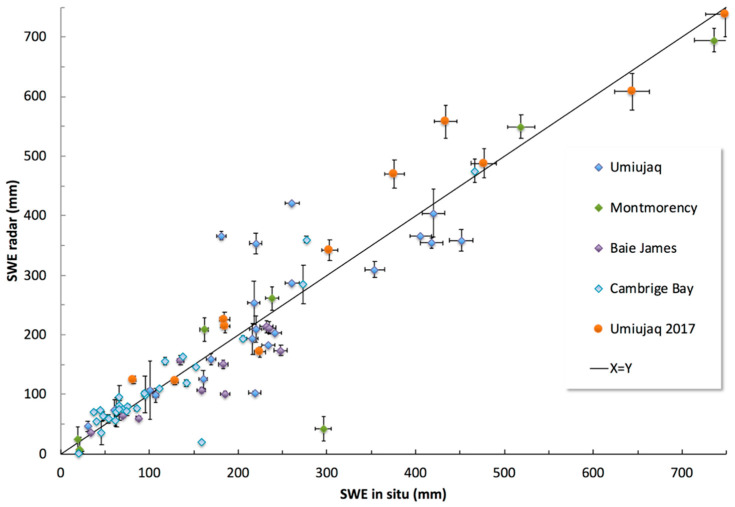
Radar-based automatic SWE estimates used with a snow depth probe compared to in situ SWE measurements (snowpit) from five field campaigns in North-Eastern Canada over dry snowpack.

A series of radar measurements over dry snowpack was compared to in situ SWE measurements over a large range of conditions (snow depth and density) in boreal forest (47° N, 18 points), subarctic taiga (54–56° N, 32 points) and Arctic tundra (69° N, 28 points) zones along a North-Eastern Canada latitudinal transect. Results are shown in Figure 13, giving an overall accuracy of 30% (RMSE of 59 mm) and relative error (Abs(Bias)/mean SWE) of 20%. Six obvious outliers (6 points with bias > 2 × mean) correspond to measurements with weak bottom peak detection amplitudes. The misdetection of these peaks was generally due to the inclination of the plate in relation to the radar position. By applying a quality control check based on a threshold on this bottom amplitude peak, we reduced the RMSE to 20% and relative error to 13%, in the range of the relative accuracy of the in situ snowpit measurement.

In conclusion, these radar-based automatic SWE retrievals, optimized with a metallic plate beneath the snowpack, were in very good agreement with in situ measurements and achieved the same RMSE. However, it is important to note that, after the first reflection at the air-snow interface (first peak), the metal plate reflector makes it possible to identify the distance from the bottom without ambiguity [32]. Without the plate, the bottom echo is sometimes not visible or very weak, requiring more sophisticated methods of signal recognition or visual checking of each measurement.

### 4.4. In Situ Density Monitoring in Antarctica

An original experiment was carried out in Antarctica with the FMCW radar to monitor, for the first time, the continuous evolution of surface snow density. The deployment was performed in 2018, starting on January 6 at the French-Italian Dome Concordia station on the East Antarctica Plateau (Dome C, 75° S, 123° E). The mean annual snow accumulation is exceptionally low at Dome C (8–10 cm/yr), with very low short-term snow depth variability in the order of a few cm. The only apparent variations in surface density are linked to wind variability, and year-to-year variability is remarkably low, except for specific short storm events [62].

**Figure 14:**
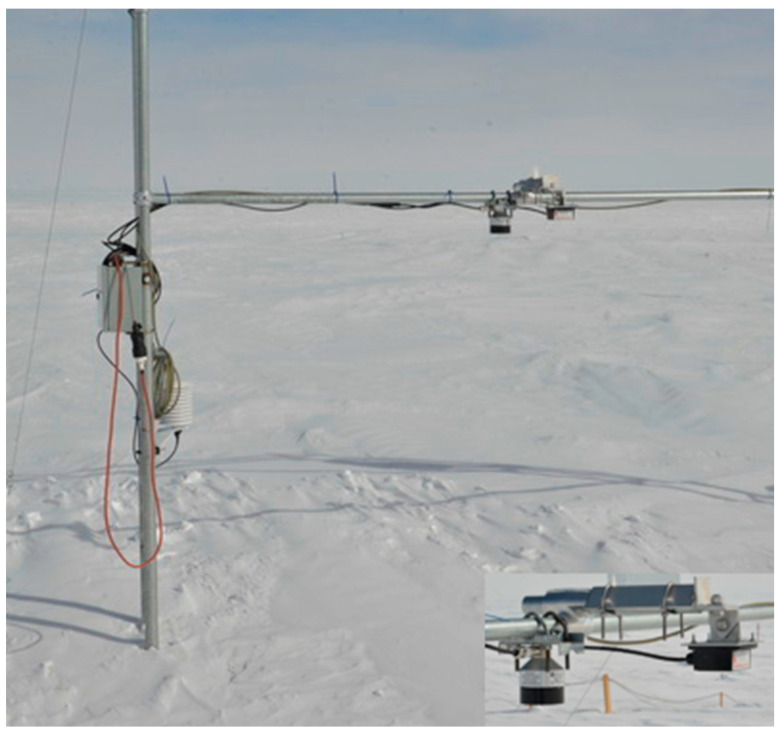
Set-up of the installation at Dome C, Antarctica. The horizontal mast was at 1.5 m above the snow. The close-up shows the ultrasonic snow depth sensor on the left, and the FMCW radar on the right.

The radar and a snow depth sensor (Campbell Scientific®, Edmonton, Canada SRAT50) were installed on a horizontal mast at 1.5 m above the snow (Figure 14), giving a radar footprint of approximately 0.64 m. In this experiment, the radar was controlled with an Arduino card because of its lower power consumption when compared to a RaspBerry Pi© controller (Raspberry Pi Foundation, 37 Hills Road, Cambridge, CB2 1NT, UK). The measurements were acquired with a time step of 10 min. Unfortunately, the system began to have operating problems on January 26 and failed when air temperature dropped to −50 °C on April 30, 2018. This failure was likely due to Arduino’s operating temperature limit. In situ density measurements were taken regularly in the vicinity of the radar spot, over the first 5 cm surface layer at five nearby sites every day. The mean air temperature during the experiment was −29 °C (from −16 to −44 °C).

**Figure 15:**
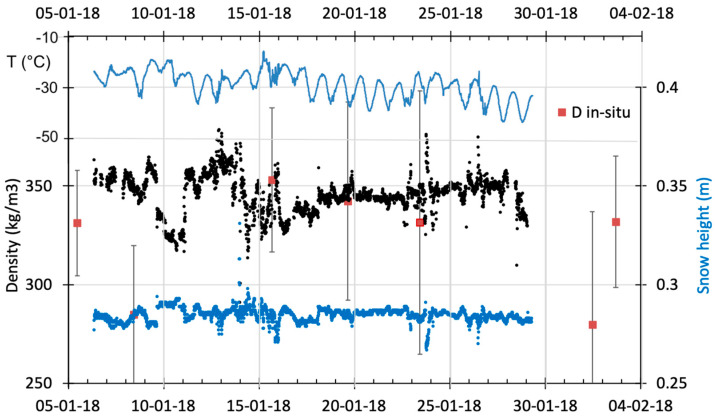
Variations of surface snow density from the FMCW radar (over the 30 cm top layer) (black points, left axis), in situ surface density measurements (red points with spatial variability), air temperature (light blue) and snow height (dark blue points, right axis) in January 2018 at Dome C (East Antarctic Plateau).

Figure 15 shows a sample of recorded values over the first month. The mean snow height variation above the plate was 28.5 ± 0.5 cm, with a variation of ± 12 cm between the maximum (34 cm) and minimum (31 cm) in the month of January. These values are slightly higher than mean surface elevation changes measured with a laser scan sensor deployed in 2015 at Dome C [62], giving 10–15 cm for the months of December and January. Differences in snow elevation could be explained by the difference in footprint size between the laser scan (10 × 10 m^2^) and the radar (less than 1 × 1 m^2^), with the former possibly smoothing frequent changes in surface shape caused by redistribution.

The mean radar-based density was 346 ± 10 kg m^−3^, ranging from 378 to 291 kg m^−3^ (Figure 15), in agreement with the measured in situ density variation, which had a range of 353 to 285 kg m^−3^ for the upper 5 cm of the snow surface during the period. The spatial variability of the five measurements per site (±42 kg m^−3^) was slightly higher than the temporal variability (±26 kg m^−3^) over the period analyzed (Figure 15). This range of variability was also measured in January 2014 along several hectometer scale transects around Dome C. It was shown that the observed surface density variations result from wind-formed features (dunes), alternating between dense/hard (434 kg m^−3^) and light/loose (372 kg m^−3^) snow areas over the first 35 cm [63]. While these observations were not made in the same year, such typical behavior is always present [62].

In conclusion, this preliminary experimental test at Dome C shows very interesting potential for continuous surface snow density monitoring. However, further work is needed to improve the robustness of the system. In the broader context of better knowledge of snow surface processes in Antarctica, such as the unknown trend of surface density change over the East Antarctic Plateau derived from satellite data [64], this approach appears to be a promising cost-effective tool for a network and to achieve year-round monitoring.

### 4.5. Continuous and Autonomous SWE Measurements 

A fixed radar station system was developed for automated continuous winter SWE monitoring and was installed in a boreal environment at Forêt Montmorency research station (NEIGE-FM) (47°19′0′′ N; 71°9′5′′ W). This snow research station is part of the World Meteorological Organization (WMO) Global Cryosphere Watch (GCW) Surface Network CryoNet (WMO ID: 71212) [5]. The set-up requirements included a precise fixed distance between the radar and the reference target (plate) on the ground, and an additional sensor to measure the snow depth as close as possible to the radar footprint. Snow depth measurements were acquired by an SR50AT sonic sensor from Campbell Scientific Canada^®^ (Edmonton, AB, Canada). The radar was placed at a height of 3 m in an assembly with the plate welded to the mast (rigid frame) to maintain a constant distance between them, even when freeze/thaw effects caused ground heaving. The radar footprint was 1.3 m, but a plate size of 0.60 × 0.60 m was enough to enable a clear bottom echo. In this experiment, the radar time acquisition frequency was set at 6 h to optimize the power consumption from its lead battery (no solar panels were used). The radar-based SWE values, processed by the in-system processor, were automatically transferred along with other data (snow depth, radar profile, air temperature—*T_air_*) every day via a cellular telephone connection. In order to validate the radar measurements, a gamma ray SWE sensor was installed at about 3 m from the radar (GMON-CS725 sensor from Campbell Scientific Canada^®^, with a footprint of 7–10 m and a time acquisition frequency of 6 h) [16,65]. Snowpit and snow core measurements were also performed throughout the winter in the vicinity of the sensors.

**Figure 16:**
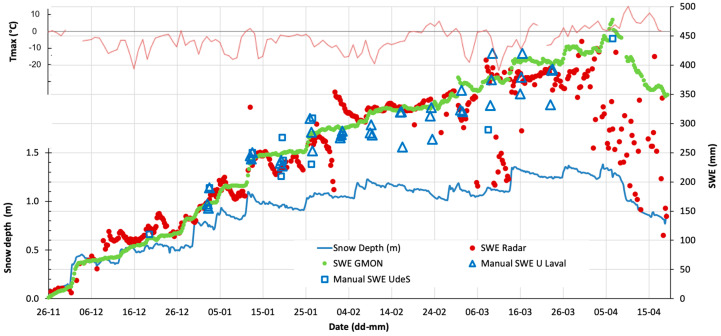
Time variations in snow depth (m) (blue line, left axis) and SWE (mm) (right axis) from the GMON sensor (green line), the radar sensor (red points) and in situ measurements (open blue squares and triangles) around the GMON-radar site at NEIGE-FM. The mean daily maximum air temperature (Tmax) (thin red line) is overlaid (upper left axis). In situ measurements were performed by two teams from Université de Sherbrooke (UdeS) and Université Laval. Measurements were made during winter 2016–2017.

Figure 16 shows a comparison of a sample time series of SWE variation retrieved from the radar and GMON sensors. Results show strong agreement between these sensors during the dry snow period, and both were in the range of in situ measurements. In situ measurements showed high variability due to spatial variation of the snowpack in the area surrounding the GMON-radar site. For the dry snow period (identified with negative mean daily maximum air temperature, red line in Figure 16), the observed radar-based SWE fluctuations can be explained by several factors: Radar acquisition is instantaneous every 6 h, while GMON has an integrated value over 6 h, thus the former captures short snow variations particularly during snowfall or strong wind events (e.g., the significant decrease in snow depth on January 29, 2017); the slight differences in location between GMON and radar positions could produce snow depth differences; and the formation of ice crust caused by wet snow refreezing on top of the snowpack after rain events. These short fluctuations can be smoothed over one or two days. Throughout the dry snow period, the SWE root mean square difference (RMSD) between radar and GMON sensors was 25.4 mm (10%), with a mean relative bias of 13.5%.

However, as soon as the snow became wet relative to the maximum air temperature, stronger attenuation in the microwave signal occurred, and the radar retrieval was biased, leading to radar-based SWE underestimation. This is clearly apparent during warm episodes (February 22 to March 1) and at the end of the season (melting onset on April 1, 2017) (Figure 16). While the melting onset could be flagged and eliminated when *T_air_* becomes positive, the retrieval algorithm for wet snow could also be improved (see Discussion). 

In conclusion, automatic radar-based SWE monitoring of dry snowpack can be achieved with common standard accuracy—typically 10–15%—with the set-up described above, particularly when combined with a snow depth sensor (ultrasonic or LiDAR) and a metallic plate on the ground to increase the snow/plate permittivity contrast. The strong limitation to the continuous winter-long use of the radar is that melting snow can be flagged with coincident temperature measurements.

## 5. Discussion

**Figure 17:**
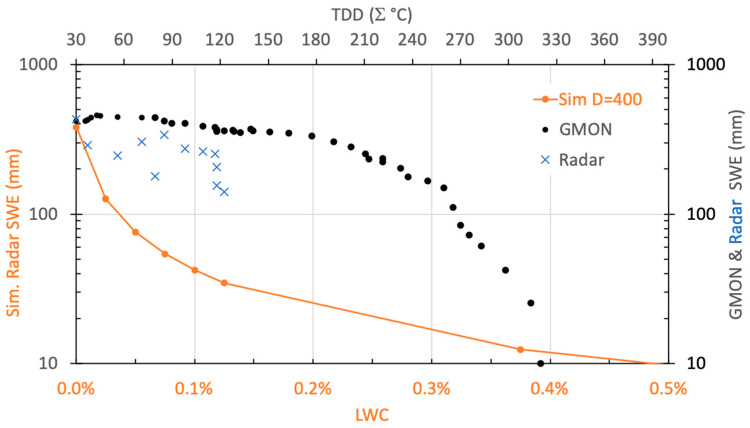
Simulated SWE (orange dots, left axis) as a function of LWC (bottom X axis), and measured SWE using GMON ((black dots) and radar (blue cross) as a function of thawing degree-days (TDD) (top X axis). The top horizontal axis starts on the melting onset. The radar was stopped on April 19, 2017 (TDD = 140 degree-days).

**Figure A3:**
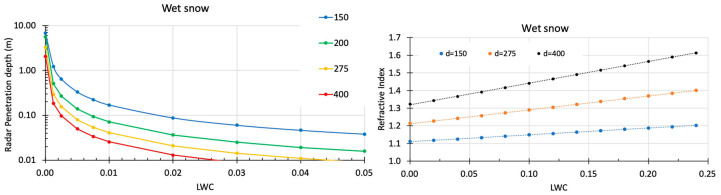
Radar penetration depth (m) (left) and refractive index vs. liquid water content (LWC) (fraction of volume) for wet snow at four densities: 150, 200, 275 and 400 kg m^−3^.

One of the main limitations of the radar is the presence of liquid water on ice or in the snowpack. Liquid water strongly absorbs the radar signal, leading to a high reflectivity at the air/water or air/wet snow interface and a weak transmissivity. When the liquid water content (LWC, in volume fraction, %) in snow increases, the effective refractive index of wet snow ($n_{sw}^{\prime })$ increases, but the two-way radar penetration depth ($\delta P_{r},$ m) decreases abruptly from 2 m (dry snow) to 0.05 m for LWC ≈0.5% at a density of 400 kg m^−3^ (Figure A3 in Appendix A). In order to analyze the radar response during the beginning of a melting period (Figure 17), we simulated SWE (orange dots) decreasing with LWC (%, bottom axis), and overlaid it with the radar-based (blue cross) and GMON-based (black dots) SWE measurements according to the thawing degree-days (TDD) (sum of positive maximum air temperature computed from the maximum measured SWE) (top horizontal axis). TDD values start at 30 degree-days corresponding to the continuous melting onset (Figure 17). No LWC measurements were available. This comparison between measured and simulated SWE during the melting period can be explained by the fact that LWC is not homogenously vertically distributed over the snowpack, as assumed in simulations. Results suggest that a threshold could be applied on radar measurements based on TDD to detect the onset of the melting period. Further studies (in progress) are needed to quantify the LWC effects on radar response and to improve early snow melting detection, i.e., LWC < 1%. Theoretical values of the wet snow refractive index and radar penetration depth as a function of LWC given in Appendix A could be useful for this purpose.

Another limitation is the formation of an ice crust resulting either from short episodes of warmth (thawing/freezing of snow surface) or from rapid refreeze after rain-on-snow events or freezing rain events. These weather episodes, which are becoming increasingly frequent with global warming, bias the radar signal retrieval. A high-density ice crust could generate a strong wave reflection and mask the bottom of the snowpack. Furthermore, internal reflectors, such as refrozen melt water, can be embedded in the ice and result in an ice lens that reduces wave propagation, thus altering the retrieval [66].

Lastly, the snowpack is usually stratified in several layers of different densities, creating a series of clearly visible echoes at each interface with a higher permittivity than the layer above (see examples in Figure 1 and Figure 12). This makes it possible to analyze the stratification of the mantle, a particularly interesting application for avalanche prediction (e.g., [32,37,44,46]). 

Within a multilayer (multi-reflectors) snowpack, the amplitude Â of the measured beat signal of the i-th interface is proportional to the magnitude $\left|r_{i}\right|$ of the i-th reflection coefficient, which also depends on the reflection coefficients of the i − 1 preceding interfaces and on the transmission loss factor $L_{i}$:

(15) $$Â\sim L_{i}\;\left|r_{i}\right|\;\overset{i-1}{\underset{k=1}{\prod }}\left(1-{\left|r_{i}\right|}^{2}\right)$$

For thin layers, reflectivity is a function of frequency due to constructive and destructive interferences. In our case, the FMCW radar operated from 23 to 25.5 GHz, i.e., in a range of wavelengths from 1.1 to 0.97 cm in snow. For layers that are much thicker than the wavelength, the reflectivity depends only on the relative dielectric contrast at the interface, while for layers which have a thickness much smaller than the wavelength, the reflectivity depends upon the layer thickness. Further analyses with special attention to frequency, absorption and propagation velocity within those multiple reflections are needed to interpret such signal features.

## 6. Summary and Conclusions

We presented a low cost, compact and lightweight (under 500 g with battery) 24 GHz FMCW radar, commercialized by IMST (IMST, 2019). Plugged into a Raspberry Pi© or Arduino controller, and with a wireless connection to a mobile phone or a handheld PC, this near-turnkey system is easy to implement and is highly adaptable for several uses. Improved distance detection was obtained by optimizing radar settings and applying simple signal processing based on finding the true maximum around the measured maximum (weighted average calculation of the distance between the maximum and its two closest neighbors). The capabilities of the FMCW radar for ice thickness and snow cover monitoring have long been known, but retrievals have rarely been automated. Here, we tested the performance and limitations of this small IMST radar in five different operational applications with different operating modes:

(1) In situ lake ice thickness measurements give a 2 cm accuracy above a 4 cm resolution limit and can measure up to 1 m of ice. The safety limit to support a man on ice is approximately 10 cm. These results were obtained assuming pure ice and for dry ice with and without a dry snow cover. However, as soon as water is present, detection is no longer possible, which is a well-known limitation of the radar system at these high frequencies.

(2) We show that this radar can be put onboard a light remotely piloted aircraft (here a Phantom 2 quadcopter) for lake ice thickness measurements from low-altitude flight (5 m). This enables surveys of, for example, unsafe thin ice and long river transects.

(3) In situ radar measurements were carried out with a joint measurement of snow depth (e.g., with an avalanche probe) to derive the mean density and the snow water equivalent (SWE) for a dry snowpack. For sets of very different snow measurements (boreal, subarctic and arctic snow), a very good overall average accuracy of 13% (RMSE 20%) was achieved by putting a metallic plate on the ground beneath the snow to increase bottom detection. Without this plate, the snow/ground interface was less detectable, which makes SWE estimation much less efficient, making more refined peak detection algorithm necessary. We showed that this radar is capable of dry SWE measurements of snowpacks up to 3 m thick (SWE up to 800–1000 mm).

(4) In the unique conditions of the Eastern Antarctic Plateau, where snow height variations are extremely low through the year (snow accumulation < 10 cm), we successfully tested continuous and automatic tracking of surface snow density over the first 30 cm. This original experiment gave a relative bias of 15 kg m^−3^ (4%) when compared to only five validation points with significant spatial variability (13%) over a period of one month. Further studies are needed to validate this experiment.

(5) The last experiment we tested was continuous and autonomous winter-long SWE monitoring with a collocated and synchronized snow depth sensor (ultrasonic or LiDAR sensor). Results show a low 13.5% bias and 25 mm RMSD (10%) for the dry snow period, compared to a gamma ray SWE sensor (GMON-CS725 sensor from Campbell Scientific Canada^®^). It must be noted, however, that since SWE data is biased by wet snow, a snowmelt detection algorithm is needed. Numerical simulations of SWE variations according to the liquid water content in snow show the high sensitivity of the radar to the melting process.

In controlled manual operating mode, we conclude that the observed capabilities of this radar in different applications and environments, including arduous climatic conditions, make this an extremely reliable system and cost-effective instrument. However, in an autonomous and continuous operating mode, unfavorable operational conditions outside the limits of radar use may occur, such as for ice and wet snow conditions. In these cases, care must be taken to avoid misinterpretation of the measured signal.

## Figures and Tables

**Table 1:** Main specifications of IMST’s sentire^TM^ Radar Module. The radar can be operated in continuous-wave (CW) radar mode (Doppler mode) or in frequency-modulated continuous-wave (FMCW) radar mode (see IMST [51] user guide for details).

Parameters	Specifications
*General*	
Modulation	CW or FMCW mode
Operating Frequency	24.25 GHz, band width B = 2.5 GHz
Discrete time-domain signal	1024 data samples
Number of Channels	1 Transmit-Channel, 2 Receive-Channels with I/Q demodulator for each channel
Data Interface	SPI *, CAN **, Ethernet (with PoE ***)
*Antenna*	
Antenna Type	Integrated Patch Antenna
Number of antennas	1 transmitter antenna and 2 receiver antennas
Antenna Characteristics	±32.5° azimuth and ±12° elevation (± 2–3°)
Antenna Polarization	linear
*Measurement*	
Measurement Range	0.6–307 m
*Operation Parameters*	
Frequency Ramp Duration (Tr)	1–100 ms
Update Rate	typically 10–200 Hz
EIRP **** Output Power	typ. 10–19 dBm (tunable)
Operating Temperature	minimum −40 °C, maximum 60 °C
*Power Supply*	
Operation Voltage	10.5–13 V, 44–54 V PoE
Standby Power	3.0 W
Operating Power	4.5 W

* In case of the Serial Peripheral Interface (SPI), an external SPI-to-USB adapter is needed to connect the radar directly to the computer; ** Controller Area Network (CAN); *** Power over Ethernet (PoE); **** Effective Isotropic Radiated Power.

**Table 2:** Hardware: Housing specification.

Parameter	Specification
Dimension (L × W × H)	98.0 mm × 87.0 mm × 42.5 mm (Housing) 114.0 mm × 87.0 mm × 42.5 mm (with Bushing)
Weight	280 g
Mounting	4 Mounting Holes (Ø 5 mm)

**Figure A1:**
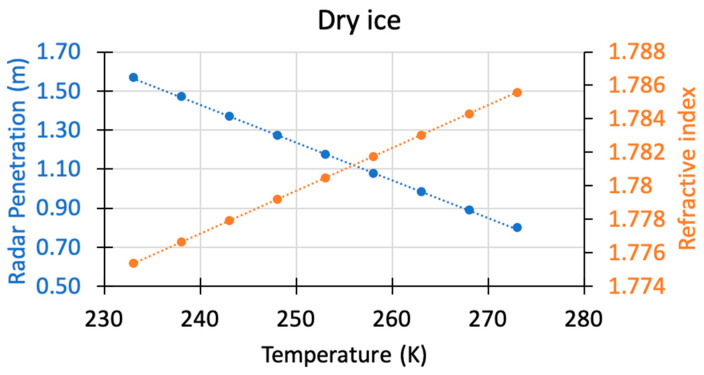
Radar penetration depth (m) (left axis) and refractive index (right axis) vs. temperature (K) for dry ice.

**Figure A2:**
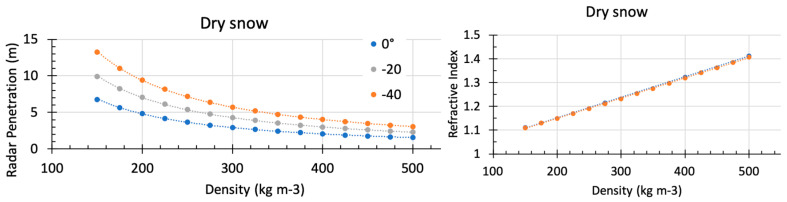
Radar penetration depth (m) (left) and refractive index (right) vs. density (kg m^−3^) for dry snow.

## Appendix A. Ice and Snow Refractive Index and Penetration Depth Values

All values and relationships are given for *f* = 24 GHz.

Ice density $\rho_{i}$ = 917 kg m^−3^

Water density $\rho_{w}$ = 1000 kg m^−3^

Dry snow density $\;\rho_{sd}$, from 150 to 500 kg m^−3^

Wet snow density $\;\rho_{sw}=\rho_{sd}+\mathrm{LWC}\;\rho_{w}\;$, valid for low LWC values (≤25%) where LWC = liquid water content in volume fraction (other formulation: $\;\rho_{sw}=\rho_{sd}\left(1-\mathrm{LWC}\right)+\mathrm{LWC}\;\rho_{w}\;$).

Wet snow volume fraction: $\nu_{sw}=\frac{\;\rho_{sd}}{\;\rho_{i}}+\frac{\;\rho_{w}}{\;\rho_{i}}LWC$

Permittivity $\varepsilon_{x}=\varepsilon_{x}^{\prime }+j\varepsilon_{x}^{\prime \prime }$, $\varepsilon_{x}^{\prime }$= real part and $\varepsilon_{x}^{\prime \prime }$ imaginary part, where $j=\sqrt{-1}$ and for ice *x = i,* water *x = w*, dry snow *x = sd* and wet snow *x = sw*

Refractive index $n_{x}=n_{x}^{\prime }+jn_{x}^{\prime \prime }$

Relationships between $n_{x}$ and $\varepsilon_{x}$ [60]:

$$n_{x}^{2}=\varepsilon_{x}\;$$

$$\Rightarrow \;{\left(n_{x}^{\prime }+jn_{x}^{\prime \prime }\right)}^{2}=\varepsilon_{x}^{\prime }+j\varepsilon_{x}^{\prime \prime }$$

(A1) $$\Rightarrow n_{x}^{\prime }{}^{2}-n_{x}^{\prime \prime }{}^{2}+j2n_{x}^{\prime }n_{x}^{\prime \prime }=\varepsilon_{x}^{\prime }+j\varepsilon_{x}^{\prime \prime }$$

$$\Rightarrow \varepsilon_{x}^{\prime }=n_{x}^{\prime }{}^{2}-n_{x}^{\prime \prime }{}^{2}\;\mathrm{and}\;\varepsilon_{x}^{\prime \prime }=2n_{x}^{\prime }n_{x}^{\prime \prime }$$

$$or\;n_{x}^{\prime }{}^{2}=\left(\frac{1}{2}\right)\left(\sqrt{\varepsilon_{x}^{\prime }{}^{2}+\varepsilon_{x}^{\prime \prime }{}^{2}}+\varepsilon_{x}^{\prime }\right)\;\mathrm{and}\;n_{x}^{\prime \prime }{}^{2}=\left(\frac{1}{2}\right)\left(\sqrt{\varepsilon_{x}^{\prime }{}^{2}+\varepsilon_{x}^{\prime \prime }{}^{2}}-\varepsilon_{x}^{\prime }\right)\;$$

It is the real part of the refractive index ($n_{x}^{\prime })$ that characterizes wave propagation, while the imaginary part characterizes absorption.

The proposed relationships were estimated with numerical permittivity values derived from simulations using the Snow Microwave Radiative Transfer Model (SMRT) code at 24 GHz [62]. All relationships had highly significant fits with very low *p* values (R^2^ ≈ 1). The linear or power law relations are given in Table A1 and shown in Figure A1, Figure A2 and Figure A3.

For example, for ice dependence with temperature (T), the refractive index varies linearly from 1.786 to 1.775 for T = 273 K to 233 K [53], respectively, and the radar penetration depth was from 0.80 m to 1.57 m.

The dry snow refractive index ($\;\rho_{sd}$) varies linearly with density from 1.111 to 1.412 for $\;\rho_{sd}$ = 150 kg m^−3^ to 500 kg m^−3^, respectively, with a negligible variation in T. The radar penetration depth ($\delta P_{r}$) of dry snow significantly decreases with density following a power law, which also varies with temperature. At T = 0 °C, $\delta P_{r}$ decreases from 6.78, 4.81, 3.26, 2.05 m for densities of 150, 200, 275 and 400 kg m^−3^, respectively. For a medium density of 275 kg m^−3^, $\delta P_{r}$ increases from 3.26 m to 4.78 and 6.38 for T = 0 °C, −20 °C and −40 °C, respectively.

The wet snow refractive index ($n_{sw}^{\prime }$) was derived using the Polder Van Santen formulation as a function of dry snow density (volume fraction) and wet ice permittivity, calculated with liquid water content (LWC, fraction of volume) (see SMRT code and references included, [67]). $n_{sw}^{\prime }$ increases with LWC and density. For LWC = 10%, $n_{sw}^{\prime }$ are 1.149, 1.289 and 1.440 for densities of 150, 275 and 400 kg m^−3^, respectively. The radar penetration depth in wet snow is very sensitive to LWC and decreases extremely rapidly. No significant fits were found. Results are shown in Figure A3 for LWC < 5% and for four densities: 150, 200, 275 and 400 kg m^−3^. For LWC = 0.125%, LWC was as low as 1.2, 0.44, 0.29 and 0.19 m for 150, 200, 275 and 400 kg m^−3^, respectively.

The SMRT code is open source and available on Github and the SMRT website [67].

**Table A1:** Relationship between the refractive index and radar penetration depth for ice, dry snow and wet snow. Fits were derived based on SMRT code permittivity values [67]. Note that some of them are unit dependent. See Figure A1, Figure A2 and Figure A3 below.

	Refractive Index (n)		Radar Penetration Depth (m)		Remarks
**Ice**	$n_{i}^{\prime }$ = 2.5555E-04 T + 1.7158		$\delta P_{r}$ = −1.9298E-02 T + 6.0610		T in K
Water	T = 0 °C	$n_{w}^{\prime }$ = 4.82626971	0		[68]
	T = −5 °C	$n_{w}^{\prime }$ = 4.46116371			
Dry snow	$n_{sd}^{\prime }$ = 8.6148E-04$\;\rho_{sd}$ + 9.7949E-01 for T = 0 °C		T = 0 °C	$\delta P_{r}$ = 3.1975E+03 $\;\rho_{sd}$^−1.2269^	$\;\rho_{sd}$ in kg m^−3^ Very slight dependence in T for $n_{sd}^{\prime }$
			T = −20 °C	$\delta P_{r}$ = 4.6162E+03 $\;\rho_{sd}$^−1.2244^	
			T = −40°C	$\delta P_{r}$ = 6.0949E+03 $\;\rho_{sd}$^−1.2224^	
Wet snow	$\;\rho_{sd}$ = 150 kg m^−3^	$n_{sw}^{\prime }$ = 0.3861 LWC + 1.1101	See Figure A3 left		$n_{sw}^{\prime }$ are for LWC ≤ 0.25 and T = 0 °C
	$\;\rho_{sd}$ = 275 kg m^−3^	$n_{sw}^{\prime }$ = 0.7892 LWC + 1.2111			
	$\;\rho_{sd}\;$= 400 kg m^−3^	$n_{sw}^{\prime }$ = 1.2268 LWC + 1.3188			

## Appendix B. FMCW Radar Simplified Link Budget

The simplified link budget for the snow-to-air reflection is defined here for a homogenous snowpack with an ideal smooth surface. The received power were estimated by the radar equation:

(A2) $$P_{RX_{i}}=P_{RX}G^{2}\frac{\lambda^{2}{\left|r_{i}\right|}^{2}}{{\left(4\pi \right)}^{3}h_{i}^{4}}\sigma$$

where G is the antenna gain, hi is the reflection distance, λ is the wavelength and σ is the radar cross-section.

We used the permittivity of dry snow density provided in Appendix A and calculated the reflection coefficients $r_{i}$ for the snow/air interface using Equation (2), expressed in dB:

(A3) $$R_{i}^{\left[dB\right]}=20\;log\left(r_{i}\right)$$

Their corresponding return losses resulted in −29 dB and −15.4 dB for snow density values of 100 kg m^−3^ and 500 kg m^−3^, respectively.

The radar cross section can be derived from the relation (A4) [69]:

(A4) $$\sigma \approx 2\pi h_{i}^{2}\left(1-cos(\frac{2\pi h_{i}^{2}tan^{2}\left(\alpha \left(h_{i}\right)\right)}{\lambda h_{i}})\right)$$

where the incidence angle $\alpha \left(h_{i}\right)$ is estimated by $tan^{2}\left(\alpha \left(h_{i}\right)\right)\approx \frac{\delta h}{2h_{i}}$ with the distance resolution δh (Equation (10)). For a mean transmitted frequency of 24 GHz and a reflection distance of 1 m, the resulting cross section is ≈12.4 m^2^. Considering the RF power between 0 dBm to 9 dBm and antenna gains G of 10 dB, the $P_{RX_{i}}$ power is –40 dB to –31 dB including the free space loss.

The total estimated budget including losses from snow reflection is therefore in the range of −69 dB (worst case) to −46.4 dB (best case). However, this link budget is valid for clear atmospheric conditions and homogeneous snowpack.
